# Role and Morbidity of Protective Ileostomy after Anterior Resection for Rectal Cancer: One Centre Experience and Review of Literature

**DOI:** 10.3390/jcm12237229

**Published:** 2023-11-22

**Authors:** Claudio Coco, Vincenzo Tondolo, Luca Emanuele Amodio, Donato Paolo Pafundi, Federica Marzi, Gianluca Rizzo

**Affiliations:** 1 U.O.C. Chirurgia Generale 2, Fondazione Policlinico Universitario A. Gemelli IRCCS, 00168 Rome, Italy; claudio.coco@unicatt.it (C.C.); donatopaolo.pafundi@policlinicogemelli.it (D.P.P.); 2 Digestive and Colo-Rectal Surgery Unit, Ospedale Isola Tiberina-Gemelli Isola, 00186 Rome, Italy; vincenzo.tondolo@fbf-isola.it (V.T.); lucaemanueleamodio@virgilio.it (L.E.A.);

**Keywords:** rectal cancer, anterior resection, anastomotic leak, protective stoma, stoma closure

## Abstract

The creation of a protective stoma is considered a valid life-saving tool, significantly reducing the effects of anastomotic leakage in terms of related morbidity, mortality, and reoperation rate. The aim of this study was to evaluate the impact of a protective loop ileostomy in terms of short- and long-term postoperative morbidity, quantifying the stoma-related complications arising after stoma creation and stoma closure and the risk of permanent stoma. From January 2009 to January 2020, 149 patients with rectal cancer treated by anterior resection and protective ileostomy were enrolled in the study. A total of 113 (75.84%) patients were preoperatively treated with neoadjuvant radiochemotherapy. A clinically relevant anastomotic leak occurred in two patients (1.34%). The postoperative stoma complication rate was 6%. According to the Clavien classification, the stoma-related complication grade was I in seven patients (4.7%) and II in two patients (1.3%). A late stoma-related parastomal hernia occurred in one patient (0.67%). In 129 patients (86.57%), it was possible to close the stoma. Postoperative complications of stoma closure occurred in 12 patients (9.3%). The stoma closure complication grade was I in seven cases (5.43%), II in two cases (1.55%), and ≥3 in three cases (2.33%). Incisional hernia was the only late complication recorded in seven cases (5.42%). The permanent stoma rate was 13.43%. A protective ileostomy has a nonnegligible complication rate, but the rate of severe complications is low. Every effort should be made to clearly identify patients in whom the risk of anastomotic leakage justifies the stoma.

## 1. Introduction

Anterior resection with mesorectal excision is considered a cornerstone in the treatment of rectal cancer. The creation of a colorectal or coloanal anastomosis represents a critical step during anterior resection due to the risk of postoperative anastomotic leakage. In the literature, the incidence of anastomotic leakage is extremely variable and ranges between 1% and 21% [1,2,3,4,5,6].

The occurrence of an anastomotic leak seems to significantly influence postoperative morbidity and mortality [2,7,8], postoperative functional outcomes [9,10], and oncological outcomes [11,12,13,14].

Considering the several negative consequences of an anastomotic leak, the creation of a stoma with the aim of diverting the faecal stream outside the abdominal cavity without passing through the anastomosis could be a lifesaving procedure to reduce the consequences of an anastomotic leak. In this context, several studies and meta-analyses have recognized the protective role of a covering loop ileostomy in reducing the rate and, especially, the clinical consequences of an anastomotic leak [15,16,17]. However, the presence of a protective stoma is associated with a nonnegligible stoma-related morbidity related to stoma creation, stoma care, and stoma closure [15]. Moreover, the creation of a temporary protective stoma predisposes individuals to a risk greater than 10% in which the stoma itself becomes permanent with a significant detrimental effect on the patient’s quality of life [15,18]. In consideration of the nonnegligible rate of stoma-related morbidity, several studies have analysed the effectiveness of alternative methods to protective ileostomy that would guarantee the same protection against the clinical effects of anastomotic dehiscence [19].

In our institution, a protective loop ileostomy was created in all rectal cancer patients submitted to anterior resection after preoperative chemoradiation therapy and in patients with a high risk of anastomotic leakage. Therefore, the aim of this study was to quantify the impact of a diverting loop ileostomy after anterior resection for rectal cancer in terms of short-term and long-term morbidity by evaluating the incidence of stoma-related complications both after stoma creation and after stoma closure and quantifying the rate of a permanent stoma.

## 2. Materials and Methods

The data on all consecutive patients treated by radical rectal resection with colorectal anastomosis and protective ileostomy for rectal cancer between January 2009 and January 2020 at U.O.C. Chirurgia Generale 2 in Fondazione Policlinico Universitario A. Gemelli IRCCS (Rome) were entered into a database and retrospectively analysed.

Inclusion criteria were as follows: age between 18 and 85 years; resectable rectal cancer (both intra- and extraperitoneal rectum, from internal anal orifice to 15 cm from internal anal orifice); anterior rectal resection with negative resection margins; defunctioning loop ileostomy caused during primary surgery; no previous colorectal surgery; no metastatic disease; and no history of inflammatory bowel disease.

All patients with endoscopic and histological diagnoses of rectal cancer were staged with CT scan and/or MRI to evaluate resectability and to establish the clinical stage of the tumour. All cases were discussed among a multidisciplinary board to establish a better treatment strategy. Patients with extraperitoneal (from internal anal orifice to 10 cm from internal anal orifice) and locally advanced rectal cancer were preoperatively treated with long-course neoadjuvant chemoradiotherapy. After 8–10 weeks from the end of CRT, patients underwent clinical restaging to evaluate the tumour response. Within 12 weeks from the end of CRT, patients underwent anterior resection with TME. Additionally, patients with nonlocally advanced low-lying rectal cancer underwent preoperative CRT. Patients with intraperitoneal or nonlocally advanced extraperitoneal rectal cancer immediately underwent anterior resection with partial or total mesorectal excision according to the tumour site. TME to the pelvic floor was performed in cases of tumours of the mid and low rectum.

Before the surgical approach was initiated, all patients underwent mechanical bowel preparation, and all patients received antithrombotic and antibiotic prophylaxis. The surgeries were performed under general anaesthesia. After anterior resection with colorectal or coloanal anastomosis, when technically possible, the anastomotic integrity was tested by an air leakage test. A drain was placed in the pelvis at the end of the procedure.

A protective loop ileostomy was created after the creation of colorectal or coloanal anastomosis in all patients considered at high risk of anastomotic leakage.

Prior to stoma reversal, the colorectal anastomosis was evaluated by digital examination, endoscopic evaluation and manometric sphincter analysis in selected cases. In case of an uneventful postoperative course, stoma closure was scheduled within 8–12 weeks from primary surgery.

The following data were collected: preoperative data (sex, age, comorbidities, tumour location, cTNM, preoperative chemo/radiation therapy); intraoperative data (surgical approach, anastomotic height, type of intestinal anastomosis, intraoperative adverse events); postoperative data during primary hospitalization (anastomotic leakage, treatment of leakage; postoperative complications and morbidity; pTNM stage; duration of hospital stay); and data from hospital stay for ileostomy closure (days until closure, duration of stay, complications).

Morbidity associated with index rectal surgery was categorized according to the Clavien–Dindo classification [20], and colorectal anastomotic leak was defined as a communication between the intra- and extraluminal compartments due to a defect in the integrity of the intestinal wall at the anastomosis, which was clinically detected during clinical examination and/or at radiological exams [21].

Postoperative stoma-related morbidity was divided into two categories: morbidity caused by stoma creation and morbidity associated with ileostomy closure. Each category was further differentiated into early complications (occurring within 30 days) and late complications (after 30 days). Additionally, stoma-related morbidity was categorized according to the Clavien–Dindo classification [20].

The primary endpoints of the study were as follows: short-term (within 30 days) and long-term postoperative stoma-related morbidity after creation of a protective loop ileostomy, short-term (within 30 days) and long-term postoperative stoma-related morbidity after closure of the protective loop ileostomy, overall rate of stoma-related morbidity of a protective loop ileostomy after anterior resection, and rate of permanent stomas.

## 3. Results

From January 2009 to January 2020, 149 patients (57 females, 38.25%; 92 males, 61.75%) with a median age of 65 years (range: 27–88 years) were enrolled in the study. The most frequent tumour sites were the middle rectum, with 66 cases (44.30%); the low rectum, with 51 cases (34.22%); and the high rectum, with 32 cases (21.48%). Of these patients, 113 (75.84%) were preoperatively treated with long-course neoadjuvant radiochemotherapy.

Anterior resection rectal surgery was performed using a mini-invasive approach (laparoscopic surgery) in 91 cases (61.07%); the remaining 58 patients (38.93%) were treated by an open approach. A colorectal anastomosis was performed with a mechanical stapler in 136 cases (91.27%), and a colo-anal anastomosis was manually performed in the remaining 13 cases (8.73%). The median distance between the anastomosis and the internal anal orifice was 2 cm (range: 0–10 cm). A protective ileostomy was performed in all 149 patients (Table 1).

Regarding the postoperative outcomes, no deaths occurred within 30 days from anterior resection rectal surgery. A postoperative complication was recorded in 35 patients, with a consequent overall early postoperative morbidity rate of 23.48%. According to the Clavien–Dindo classification, the severity of postoperative complications was grade ≥ III in five cases (3.35%), with a rate of reoperation within 30 days of 2.01% (three patients). A clinically relevant anastomotic leak occurred in two patients (1.34%), and both patients needed emergency surgical reoperation. Other postoperative complications were postoperative ileus in 9.4% of cases (14 patients); in three cases (2%), postoperative ileus was referred to as a stoma complication (difficult emptying). Other postoperative complications related to primary surgery were bleeding requiring blood transfusion in 6.04% of patients, cardiac complications in two patients and pelvic abscess in one patient. All these complications occurred within 30 days of surgery (Table 2). Regarding stoma, the most frequent complications, in addition to postoperative ileus, were severe dehydration requiring hospital readmission, which occurred in three patients (2.01%), and superficial mucocutaneous separation, which occurred three patients (2.01%). No stoma-related complications needed surgical reintervention. According to the Clavien classification, postoperative stoma-related complications were grade I in seven patients (4.69%) and grade II in two patients (1.34%). All previous stoma-related complications occurred within 30 days of stoma creation, and the overall rate of early stoma-related complications after stoma creation was 6.04% (Table 2).

The median postoperative hospital stay after primary surgery of patients with stoma creation was 7 days (range: 6–23 days). Late stoma-related complications occurred in only one patient (0.67%) and was a parastomal hernia. Therefore, the overall rate of stoma-related complications after stoma creation was 6.71% (Table 2).

In 129 patients (86.57%), it was possible to close the stoma. In 20 cases, it was not possible because of a high risk of faecal incontinence (evaluated with anal manometry) in 12 patients (8.05%), metastatic disease in 6 cases (4.03%), and unsolved anastomotic leak in 2 cases (1.34%). Therefore, the rate of definitive stoma after anterior resection and protective stoma was 13.42%. The median time interval between stoma creation and stoma closure was 77 days (range: 28–419). The anastomosis was mechanical in 87 cases (67.44%) and manual in the remaining 42 cases (32.56%). No deaths occurred within 30 days of stoma closure. Postoperative complications within 30 days of surgery occurred in 12 patients (9.30%): postoperative ileus in six patients (4.65%), requiring surgery in two patients (1.5%); surgical wound infection in three patients (2.33%); intestinal bleeding in two patients; and anastomotic leakage in one patient (0.78%) requiring reoperation. According to the Clavien classification, the grade of postoperative complications was I in seven cases (5.43%), II in 2 cases (1.55%), and ≥3 in three cases (2.33%). The overall rate of early postoperative complications after stoma closure was 9.30%. The median postoperative hospital stay after stoma closure was 5 days (range: 4–15). Thirty days after stoma closure, the only complication related to stoma closure was incisional hernia, which was recorded in seven cases (5.42%). The rate of postoperative complications after stoma closure was 14.72%. The cumulative overall rate of stoma-related complications after stoma creation and stoma closure was 19.46% (Table 2).

## 4. Discussion

Anterior resection with mesorectal excision represents the gold standard in the treatment of rectal cancer. The creation of a colorectal anastomosis at various heights is considered the most critical technical step due to the potential risk of anastomotic leakage.

In the literature, the incidence rate of an anastomotic leak ranges between 1–21% [1,2,3,4,5,6]. In a large systematic review of 71 studies and 24,288 rectal cancer patients treated by anterior resection, the reported pooled overall median rate of anastomotic leakage was 8.58% (range: 1.22–20.50%) [1]. The large variability in the range of incidence of anastomotic leakage may be due to a nonunique definition of anastomotic leakage before 2010. According to the International Study Group of Rectal Cancer as established in 2010, a unique definition of anastomotic leakage after anterior resection is as follows: anastomotic leakage is the communication between the intra- and extraluminal compartments due to a defect in the integrity of the intestinal wall at the anastomosis between the colon and rectum or the colon and anus [5].

The occurrence of an anastomotic leak seems to negatively influence the postoperative outcome both in terms of postoperative morbidity and mortality than in terms of long-term oncologic and functional outcomes.

The occurrence of an anastomotic leak causes a chain reaction of immediate clinical consequences, such as intraabdominal or pelvic abscess, peritonitis, colocutaneous fistula, and sepsis, with consequences of a longer hospital stay and increased in-hospital morbidity and mortality [8]. In the large retrospective analysis of Kang et al., in 72,055 patients who underwent elective anterior resection for rectal cancer, the rate of anastomotic leakage was 13.68%. Patients who experienced an anastomotic leak had a higher rate of in-hospital mortality (1.78% vs. 0.74%) and postoperative complications, in terms of postoperative ileus (80.72% vs. 8.11%), wound infection (15.73% vs. 2.97%), respiratory failure (7.41% vs. 2.37%), urinary tract infection (7.61% vs. 2.93%), pneumonia (5.02% vs. 1.93%), deep-vein thrombosis (1.12% vs. 0.43%), and cardiac complications (3.60% vs. 1.95%), with a consequently longer mean hospital stay (14 vs. 7 days) and higher mean total charges ($93,110 vs. $51,413) [2].

Several studies have analysed the role of anastomotic leakage in worsening oncologic outcomes. In a pooled analysis of 5187 patients from major randomized controlled trials on rectal cancer, patients with anastomotic leakage had a worse 5-year overall survival rate than patients without anastomotic leakage (66.4% vs. 74.4%), and this significant difference was maintained after excluding patients who died within 90 days from surgery (71.5% vs. 75.5%) [11]. A subsequent meta-analysis of 21,902 patients also showed a significant correlation between anastomotic leakage and local recurrence, suggesting a significant impact of leakage on long-term cancer-specific survival [13]. In the CAO/ARO/AIO-94 German Rectal Cancer Trial, anastomotic leakage occurred in 86 of 579 (14.9%) rectal cancer patients, and anastomotic leakage was associated with worse 10-year overall survival (51 versus 65.2%; *p* = 0.020) [14]. Analogous evidence was found in the analysis of the COLOR II study group; exclusively in rectal cancer patients experiencing an anastomotic leak, the rates of local recurrence (13.3% vs. 4.6%; *p* = 0.005) and disease-free survival at 5 years (53.6% vs. 70.9%; *p* = 0.006) were significantly worse than those in patients without the occurrence of anastomotic leak [12]. Moreover, the anastomotic leak and, in particular, the abdominal and pelvic consequences of an anastomotic leak seem to negatively influence the postoperative functional results, especially the evacuation and continence function, as demonstrated in our previously published study on 100 rectal cancer patients [9].

Several factors influence the occurrence of an anastomotic leak. In the large meta-analysis of Kang et al., weight loss, malnutrition, fluid and electrolyte disorders, male sex, and the occurrence of postoperative complications (such as postoperative ileus, wound infection, respiratory/renal failure, urinary tract infection, pneumonia, deep vein thrombosis and myocardial infarction) were established as factors independently related to the occurrence of anastomotic leakage [2]. Another meta-analysis underlined the importance of obesity (BMI higher than 25), anaesthesiologic risk (ASA score > 2), large tumour (larger than 5 cm), and preoperative chemoradiation therapy as prognostic factors significantly related to the occurrence of an anastomotic leak [22]. In the last 30 years, several studies have underlined the role of neoadjuvant chemoradiation therapy, which plays a significant role in terms of local control and sphincter- and organ-sparing surgery [23] but seems to significantly increase the risk of colorectal anastomotic leak [5].

Considering the several negative consequences of the anastomotic leak and considering the risk factors increasing the risk of anastomotic leak, several studies have analysed the role of a protective stoma in preventing the occurrence of an anastomotic leak and in protecting against its clinical consequences. In 2010, a Cochrane systematic review of six randomized controlled trials demonstrated that the use of protective stoma significantly reduced the rate of anastomotic leakage and the rate of postoperative surgical reoperation, without any reduction in the risk of postoperative mortality [16]. The protective role of covering stoma in reducing anastomotic leak and reoperation rate was also reported in a meta-analysis of 11 studies and 5612 patients by Wu et al. [23]. Recently, a high-quality meta-analysis of eight randomized controlled trials and 892 rectal cancer patients who underwent anterior resection further emphasized the protective role of stoma diversion in reducing the rate of anastomotic leakage and the rate of postoperative reoperation [8]. The independent role of a protective stoma in preventing an anastomotic leak was also reported by large studies on the risk factors for anastomotic leak [17,24]. However, as reported in a recent cross-sectional nationwide study of 6330 patients, the adoption of mini-invasive approaches, such as laparoscopic and robotic surgery, seems to reduce the necessity of protective stoma at a range between 29% and 42% of rectal cancer patients underwent to surgery [25].

In our institution, a protective loop ileostomy was created in all rectal cancer patients who underwent anterior resection after preoperative chemoradiation therapy and in patients with a high risk of anastomotic leakage due to the height of anastomosis and the comorbidities that the patient had. In the series reported in the study, the rate of clinically evident anastomotic leakage in patients with a covering stoma after anterior resection was 1.34%.

However, the creation of a loop ileostomy is associated with a nonnegligible rate of stoma-related complications due to the creation and closure of the stoma, which significantly affects the quality of life of stoma patients. The rate of overall stoma-related morbidity after defunctioning loop ileostomy ranges between 5% and 100%, and complications can be divided into major, potentially requiring a surgical reoperation such as stenosis, small bowel obstruction, retraction, necrosis, prolapse, stricture, fistula, and parastomal hernia, and minor, such as dermatitis, electrolyte imbalance, and dehydration from high stoma output, which may often necessitate the early closure of the stoma [15]. This nonnegligible rate of stoma-related morbidity, due to several factors depending on patient comorbidities and surgical factors, has meant that several scientific societies have produced guidelines on stoma care to prevent, reduce, and correctly treat the possible onset of stoma-related complications, reducing their impact on the quality of life of these patients [26]. Moreover, some series have evaluated the effectiveness of alternative methods to loop ileostomy in preventing the incidence of anastomotic leakage and reducing its clinical consequences. The use of transanal drainage tubes has been proposed as an alternative, and recently, a meta-analysis on six studies (two randomized) and 735 patients has been published; the meta-analysis did not show any statistically significant differences between rectal tubes and diverting stoma in any outcomes, suggesting the noninferiority of rectal tubes compared with loop ileostomy [19].

The aim of this study was to evaluate the impact of a diverting stoma in terms of morbidity during construction and after closure and to evaluate the risk of a permanent stoma. The rate of overall stoma-related morbidity after construction was 6.71%, and the most frequent complications were severe dehydration requiring hospital readmission (2.01%), postoperative ileus (2.01%), and mucocutaneous separation (2.01%). Most complications related to loop ileostomy occurred within 30 days from its construction, and no events needed surgical reoperation for resolution. This morbidity rate can be considered acceptable if compared with existing data in the literature.

After a median time of 77 days (range 28–419 days), 86.57% of patients underwent stoma closure. Therefore, in 13.43% of patients in whom a protective loop ileostomy was created, it was not possible to close the stoma. The rate of definitive stoma recorded in the present study is in the range of 0–19% reported in the literature. The most frequent cause of failed closure of stoma in this series (8.05%) was the request of the patient due to the high risk of faecal incontinence after stoma closure; in six cases, the disease progressed and the patient needed immediate chemotherapy; in the two cases that had the anastomotic leak, it was not possible to close the loop ileostomy due to unsolved anastomotic problems. The failed closure of a protective stoma should be considered a stoma-related problem after stoma construction, especially considering the psychological aspects and negative effects on the patient’s quality of life and considering the risk of developing late stoma-related complications such as mechanical problems, dehydration, and psychosexual problems [18].

The median time of 77 days for stoma closure seems to be in the range of the median time of 8–12 weeks reported in the literature. A delayed time to stoma closure is a debated factor that seems to increase stoma-related morbidity and the functional results and severity of low anterior resection syndrome. In the meta-analysis of Vogel et al. (including 11 studies and 1400 patients), a longer time to stoma closure was associated with a higher risk of major LARS, and the mean difference in time to closure between the major LARS group and the no LARS group was 2.39 months [27].

The protective stoma-related morbidity must necessarily consider the postoperative morbidity related to the surgical procedure of stoma closure. In a review by Kaidar-Person, the overall morbidity after reversal of a temporary loop ileostomy ranged from 2% to 33%, and the most frequent complications reported in the literature were wound infections (0–18.3%), small bowel obstruction (0–15%), anastomotic leak (0–8%), entero-cutaneous fistulae (0.5–7%), and incisional hernias in the previous stoma site (0–12%) [10]. In a more recent systematic review of 48 studies (6107 patients) by Chow et al., the overall rate of postoperative complications after loop ileostomy reversal was 17.3%, with a postoperative mortality rate of 0.4%. The most frequent adverse events were small bowel obstruction (7.2%) and wound infection (5.0%); moreover, 3.7% of patients needed a laparotomy at the time of ileostomy closure [28].

The short-term morbidity related to stoma closure recorded in this series was 9.3%, with the need for reoperation in three patients (2.33%; two for small bowel obstruction and one for anastomotic leakage). Regarding long-term morbidity, seven patients (5.42%) experienced incisional hernia at the stoma site. The overall rate of postoperative complications after stoma closure of 14.72%, which even if not negligible, can be considered in line with the rate of morbidity reported in the literature.

Our retrospective series resulted in an overall rate of stoma-related morbidity, both after stoma creation and after stoma closure, of 19.46%, a rate in range with existing data in the literature but not negligible, especially if we consider that many covering loop ileostomy procedures could be considered not useful if we take into account the rate of anastomotic leakage. On the other hand, if an anastomotic leak occurs, the consequence of this leak in the pelvis and in the abdomen can be a life-threatening event, significantly affecting the functional results and the quality of life of the patients. Therefore, ideally, a protective stoma should be performed only in patients with a high risk of anastomotic leakage, and the creation of a score or a nomogram evaluating the risk of anastomotic leakage after anterior resection could reduce the number of unnecessary protective loop ileostomies in patients with a low risk of anastomotic dehiscence. Recently, a nomogram for the prediction of anastomotic leakage after anterior resection was created based on a multivariate analysis of 1995 patients. A nomogram was created with several factors related to anastomotic leakage, such as male sex, diabetes, neoadjuvant therapy, tumour distance from the anus verge < 5 cm, tumour size ≥ 5 cm, and blood loss > 50 mL, but this model still requires validation by prospective studies [3].

## 5. Conclusions

A protective ileostomy has a nonnegligible complication rate, but the rate of severe ileostomy is low. So, even if the consequences of anastomotic dehiscence could put the patient’s life at risk, a percentage of protective ileostomy should probably be avoided by classifying patients according to the presence of risk factors for anastomotic leak. The role of nomograms for prediction of anastomotic leakage, reducing the rate of protective ileostomy if unnecessary, has to be evaluated.

This aspect is particularly important considering that the consequences of an anastomotic dehiscence without protective ileostomy can put a patient’s life at risk. Every effort should be made to clearly identify patients in whom the risk of anastomotic leakage may be the cause of the stoma.

## Figures and Tables

**Table 1:** Baseline characteristics of patients enrolled in the study.

Variables	Number (%)
Number of patients	149
Sex (Male vs. Female)	92 (61.75%)–57 (38.25%)
Median age (range)	65 years (range: 27–84 years)
Tumour site (High vs. Middle vs. Low Rectum)	32 (21.50%)–66 (44.28%)–51 (34.22%)
Neoadjuvant Treatment	113 (75.84%)
Surgical approach (LPS vs. OPEN)	91(61.07%)–58 (38.93%)
Median distance of anastomosis from anorectal ring	2 cm (range: 0–10 cm)
Anastomosis technique (Manual vs. Mechanics)	13 (8.72%)–136 (91.28%)

**Table 2:** Postoperative complications after anterior resection and creation of a protective loop ileostomy and after protective loop ileostomy closure.

Outcomes	Number (%)
Short-term postoperative mortality after primary surgery	0 (0%)
Short-term postoperative morbidity after primary surgery	35 (23.48%)
Clinically relevant anastomotic leak	2 (1.34%)
Grade ≥ 3 postoperative morbidity after primary surgery	5 (3.35%)
Reoperation rate within 30 days after primary surgery	3 (2.01%)
Short-term stoma related morbidity after stoma creation:	9 (6.04%)
Ileus	3 (2.01%)
Severe dehydration requiring hospital readmission	3 (2.01%)
Mucocutaneous separation	3 (2.01%)
Median postoperative hospital stay after primary surgery	7 days (range: 6–23 days)
Long-term stoma related morbidity after stoma creation:	1 (0.67%)
Parastomal hernia	1 (0.67%)
Overall stoma-related complications after stoma creation	10 (6.71%)
Short-term postoperative mortality after stoma closure	0 (0%)
Stoma reversal rate–Permanent stoma	129 (86.57%)–20 (13.42%)
Median time to stoma closure	77 days (range: 28–419 days)
Short-term postoperative morbidity after stoma closure:	12 (9.30%)
Postoperative ileus	6 (4.65%)
Surgical wound infection	3 (2.33%)
Intestinal bleeding	2 (1.55%)
Anastomotic leak	1 (0.78%)
Grade ≥ 3 postoperative morbidity after stoma closure	3 (2.33%)
Reoperation rate within 30 days after stoma closure	3 (2.33%)
Median postoperative hospital stay after stoma closure	5 days (range: 4–15 days)
Long-term stoma related morbidity after stoma closure	7 (5.42%)
Incisional hernia	7 (5.42%)
Overall stoma-related complications after stoma closure	19 (14.72%)
Cumulative overall rate of all stoma-related complications	29 (19.46%)

## Data Availability

Data are unavailable due to privacy restrictions.
